# Looking for the Last Universal Common Ancestor (LUCA)

**DOI:** 10.3390/genes3010081

**Published:** 2012-01-09

**Authors:** Minna Koskela, Arto Annila

**Affiliations:** 1 Department of Biosciences, Viikinkaari 1, FI-00014 University of Helsinki, Finland; E-Mail: minna.m.koskela@helsinki.fi; 2 Institute of Biotechnology, Viikinkaari 1, FI-00014 University of Helsinki, Finland; 3 Department of Physics, Gustaf Hällströmin katu 2, FI-00014 University of Helsinki, Finland

**Keywords:** evolution, geodesic, intractability, sequence alignment, the principle of least action, universal ancestor

## Abstract

Genomic sequences across diverse species seem to align towards a common ancestry, eventually implying that eons ago some universal antecedent organism would have lived on the face of Earth. However, when evolution is understood not only as a biological process but as a general thermodynamic process, it becomes apparent that the quest for the last universal common ancestor is unattainable. Ambiguities in alignments are unavoidable because the driving forces and paths of evolution cannot be separated from each other. Thus tracking down life’s origin is by its nature a non-computable task. The thermodynamic tenet clarifies that evolution is a path-dependent process of least-time consumption of free energy. The natural process is without a demarcation line between animate and inanimate.

## 1. Introduction

The quest for the last universal common ancestor (LUCA) roots back to the much-quoted closing paragraph of On the Origin of Species where Charles Darwin infers: “…that probably all the organic beings which have ever lived on this earth have descended from some one primordial form, into which life was first breathed.” The notion of common origin is even more motivated today since biochemical, molecular biology and genetic studies have disclosed that apart from similarities in phenotypes, many molecular characteristics of life are also universal. Most notably, the same metabolites, proteins and nucleic acids (including their specific handedness) are found throughout nature [1]. Also, the genetic code is next to identical for all life forms [2]. Most importantly, similarities in genomic sequences across life’s major branches imply universal common ancestry [3]. This conclusion about the common origin seems not to be obscured by lateral gene transfer [4] or by convergent evolution to sequence similarity [5]. Even if life had emerged multiple times, LUCA has been regarded as an outcome of convergent evolution, e.g., as a single recombining population of early organisms [6] or as the only one species that survived in the course of early evolution [7]. The probability of common ancestry has been considered by statistical tests to be substantially higher than that of multi-ancestor models [8]. However, likelihood analyses may be illusory in proving the hypothesis [9], and at least ambiguous in constructing one tree of life [10]. 

Though inconclusive, the body of evidence for the common descent of all life seems overwhelming. However, we wish to point out that the notion of a single common ancestor is conceptually confusing since it places some one individual organism on a special pedestal. It is as if LUCA were the first one to depart in some significant way from its ancestors to be crowned as the earliest living being. To make such a stark contrast between animate and inanimate should not be regarded merely as a logical lapse that could be patched up by extending the notion of LUCA to an ancient community of organisms [11] or even to a primordial class of biomolecules, but the search for a last common universal ancestor indicates impaired understanding of what life actually is and how it came about [12,13].

## 2. Evolution as a Natural Process

To call living beings thermodynamic systems is nothing new [14], yet to claim that evolution is nothing but a thermodynamic process seems to cock the eyebrows of many. The modern evolutionary synthesis, by emphasizing the genetic mechanisms of evolution, narrows thinking from what evolution actually is and distracts attention from comprehending what in fact powers evolution and what is the profound basis of natural selection. 

We argue here for the thermodynamic thinking of evolution by reminding that according to physics everything that exists is associated with energy. Thereby nothing, irrespective of molecular, genetic or any other mechanism, can escape the thermodynamic imperatives that force all systems toward stationary-state in their environment. Hence evolution is regarded as any other process being driven by a motive force, which in general terms is a difference in energy of any kind. Thus, a difference between the energy of insolation and the energy in chemical potentials of molecular species [15] will drive chemical reactions on Earth. Energy flows from heights to lows when substrates transform to products. The flows of energy themselves will naturally select to channel via mechanisms, not only molecular mechanisms but any kind of machinery, that will consume driving forces in least time [16]. The selection criterion, when given in terms of physics, is not ambiguous but the least-time consumption of free energy is the unequivocal attribute of the fittest [17]. In the same way as a river bed will run dry when water breaks a more effective channel, so will a suboptimal system lose its supplies for a more effective system of species. The least-time consumption of free energy is the natural bias of evolution. It is not a deterministic but holistic imperative because evolution, as it goes, will alter its surroundings and thereby affect its own course. Hence forces of phylogenesis are physical. 

It is worth to emphasize that the physical criterion of natural selection does not determine the course of evolution because evolution itself will change the surrounding conditions which in turn will require incessant re-evaluation of the least-time path. In other words, it is not the heterogeneity and complexity of a system as such that would make its evolution intractable, but the non-computable character of natural processes manifests itself already in the case of three bodies [18]. When there are two or more alternatives to consume free energy, the natural process becomes intractable since the process itself will affect its driving forces. Undoubtedly the heterogeneity and complexity contribute to the computational challenge by providing various repositories of bound energy as well as numerous combinations of pathways and mechanisms to consume differences in energy. As a result the complex system may display at same time for a given sub-process both parallel and anti-parallel flows of energy. In other words, in nature where everything depends on everything else, some supplies pile up while others are consumed. The co-gradient and contra-gradient processes are customarily modeled, e.g., by Lotka-Volterra equations, but accurately contained in the equation of evolution [19]. The net absorption or emission of energy from the system to its surroundings displays the quest to attain stationary state in the respective surroundings. The overall net dissipation stems from the universal process that aims at attaining a stationary state with respect to “the zero-density surroundings”, *i.e.*, the heat death. The same thermodynamic imperative, however, will force diverse thermodynamic potentials to pile up and adjust to each other, when surroundings are rich in energy such as here on Earth that enjoys insolation. 

This old, general formulation of evolution [20] is not only valid for biological processes. When given by Newton’s 2^nd^ law **F** = d**p**/d*t*, it is recognized to hold for any other natural process. Evolution, when understood as a thermodynamic process, is not a random (stochastic) process at any level of natural hierarchy, but it will keep on directing its momentum **p** = *m***v** along the resultant force to diminish energy differences as soon as possible. The change in momentum d**p**/d*t* = *m***a** + **v**d*m*/d*t* involves not only acceleration *m***a** that balances the force within the system, but also the surroundings in terms of dissipation of energy d*E*/d*t* in the form of a change in mass d*m*/d*t* = d*E*/*c*^2^d*t* to the free space defined by the squared speed of light *c*^2^ [17]. In other words, although an evolving system may be subject to severe tension due to numerous non-parallel and even anti-parallel forces within the system as well as with respect to the surroundings, it will processes along the resultant force. Also the system-bound energy, *i.e.*, heat capacity, is a supply that will resist imposed changes. It will manifest itself as memory of the past trajectory. For example, the genome of an organism houses instructions of the processes that were at least in the past circumstances effective in consuming free energy. Accordingly organisms tend to proceed along the previously established paths of free energy consumption just as cultures tend to follow their traditions in hopes of making the best use of their characteristic circumstances [20].

The evolutionary equation [16] has also been known for a long time in its open, integral form as the principle of least action [21]. It says that the flows of energy of any kind will track down from heights to lows along the paths of least time. Over eons these paths of kinetic energy [22] have diverged for maximal dispersal of energy. The resulting diversity is commonly depicted as a branching phylogenetic tree.

## 3. Inherent Intractability

The present-day parlance about evolution using only biological lingo does not make use of the generality and rigor that is available when evolution is described by general terms in its entirety and every detail as a probable physical process [23] of least-time free energy consumption. The concept of energy can be assigned to everything that exists. Consequently the thermodynamic formalism will ensure the conservation of quanta irrespective of mechanistic details and specific circumstances. Notably, when evolution is given by the mathematical equation of motion [16,17,19], it can be analyzed to realize that the path-dependent process cannot be solved. The paths of evolution cannot be predicted forwards or traced backwards with certainty since the flows of energy and the differences of energy as their driving forces cannot be separated from each other [16,17,19]. Since a natural process is itself changing the surrounding conditions due to dissipation, the boundary conditions keep changing. This is the fundamental reason why evolution is intractable when there are alternative paths to consume free energy. In plain language, this means that when a species emerges with a new characteristic, whether acquired by a mutation or new experience, its altered behavior [24] will in turn exert forces on other species. According to thermodynamics evolution at all levels of natural hierarchy [25] is a process that is itself carving the energy landscape and thereby directing its own course [26,27]. There is no algorithm that could circumvent this innate character of non-computability [28]. 

## 4. Ambiguous Alignments

Evolution is also intractable at the genomic level since information is physical too [29]. Ambiguity about origin arises at the points of speciation where, mathematically speaking, derivates of evolutionary trajectories are inexact due to quantized dissipation [30]. In other words, information as free energy [29] is invariably lost during evolution. Hence the past paths can no longer be traced with certainty. 

The non-deterministic nature of phylogenesis profoundly troubles attempts to align for ancestors. To derive a cladogram even from a set of complete genome sequences of contemporary species is inherently a non-computable task [28]. The non-computability manifests itself when searching for the optimal alignment of one particular segment, because a positioning will affect the already adopted alignments of other segments. That is, the computational problem keeps changing when it is being solved [31], which is familiar from many other hard problems [32] such as protein folding [33]. Therefore, the calculated lineages, ranked in terms of probabilities, depend on the particular set of aligned sequences, and no alignment can be announced as the actual. The search for the optimal alignment is the search for the least-time path [34], but when the geodesic keeps changing by the search itself, it obviously cannot be determined unambiguously.

Aligning for ancestry is in computational terms an NP-hard problem [35], which means that increasing the number of sequences to be aligned causes an exponential growth in the computation time needed to solve the problem. Circular reasoning is also a characteristic of sequence alignments; sequences are aligned to build a tree, but to begin with the alignment itself needs a tree. The challenge is to value diverse alignments and obtained trees [36] without having a firm deterministic basis. Even the most effective algorithms [37] when modeling evolution, e.g., as if it were a Markovian process [38,39], cannot remove the principle problem of intractable past [28]. Despite its non-computable character, evolution is not a random, stochastic process, but a physical process that directs along paths where free energy will be consumed in the least time. Emergence of biota itself is impressive evidence that circumstances, often referred to as boundary conditions, have changed when a huge amount of free energy from insolation has been bound to the system. Mathematically speaking, neither probabilities are invariant nor is there an ultimate norm in sight. Therefore, deterministic algorithms and unitary transformations are powerless to reconstruct phylogenesis precisely. 

Moreover, when evolution is expressed in general thermodynamic terms, its mathematical analysis reveals, why populations of species from genes to galaxies display skewed, nearly lognormal distributions [40,41] as well as why evolution proceeds by punctuations and stasis [42] and why power laws are ubiquitous characteristics of both animate and inanimate processes and structures [18,43,44]. Since the least-time consumption of free energy as the universal criterion of natural selection renders evolution as a comprehensive and comprehensible process, the reference [45] “Nothing in biology makes sense except in the light of evolution.” is in place.

## 5. Conclusions

When evolution is understood as a thermodynamic process of least-time free energy consumption, it follows that phylogenetic analyses, as done using genetic information, will not be able to determine common ancestors. Moreover, when looking for LUCA, there is an inadvertently in-built impression that animate and inanimate would somehow be distinct from each other. This obscures comprehension of the principle of abiogenesis. Also the idea of aligning for a common ancestor is unintentionally a reductionist approach, but contemporary characters cannot be reduced one-to-one to primordial qualities because energy is not a constant of evolution. The modern evolutionary synthesis, by its gene-centered paradigm, narrows the view away from the general physical principle of evolution and thereby prevents obtaining a holistic understanding of nature as a unity.
